# Association between muscle strength, upper extremity fatigue resistance, work ability and upper extremity dysfunction in a sample of workers at a tertiary hospital

**DOI:** 10.1186/s12891-021-04256-y

**Published:** 2021-06-01

**Authors:** Thaís Marques Fifolato, Heloísa Correa Bueno Nardim, Ester Rodrigues do Carmo Lopes, Karen A. Kawano Suzuki, Natalia Claro da Silva, Felipe de Souza Serenza, Marisa C. Registro Fonseca

**Affiliations:** 1 Post Graduation Program in Rehabilitation and Functional Performance, Ribeirão Preto Medical School, University of São Paulo, Ribeirão Preto, SP Brazil; 2 Medical School of Ribeirão Preto, University of São Paulo, Ribeirão Preto, SP Brazil; 3 Ribeirão Preto Medical School, University of São Paulo, Ribeirão Preto, SP Brazil; 4 Department of Health Sciences, Ribeirão Preto Medical School, University of São Paulo, Bandeirantes Av 3900, Ribeirão Preto, SP 14049-900 Brazil

**Keywords:** Upper extremity, Muscle strength dynamometer, Work capacity evaluation, Chronic pain

## Abstract

**Background:**

Upper extremity musculoskeletal disorders negatively affect ability to perform activities of daily living, self-care and work. Therefore, outcome measurements that address muscle strength, fatigue resistance, functionality and work physical capacity must be defined to assess and plan specific actions to minimize them.

**Objective:**

To investigate the association of upper extremity muscle strength with upper extremity fatigue resistance, work ability and upper extremity dysfunction in a sample of workers from a tertiary hospital.

**Methods:**

Shoulder and elbow isokinetic strength were assessed by Biodex System 4™, isometric hand grip by JAMAR™, upper extremity fatigue resistance by Functional Impairment Test Hand and Neck/Shoulder/Arm (FIT-HaNSA), ability to work by the Work Ability Index and upper extremity dysfunction by the Quick-Disabilities of the Arm, Shoulder and Hand QuickDASH-Br questionnaire. The Nordic questionnaire and Numeric Pain Rating Scale (NPRS) were used for pain description. The associations were analysed by Spearman’s correlation coefficient (rho) (*p* < 0.05).

**Results:**

Twenty-seven participants: 59.2% women; mean age 46 years old; 70.3% obese/overweight; 62.9% active with predominantly dynamic muscle contraction work. Besides predominance of good to moderate work ability (81.4%) and comorbidities (37%), all participants had symptoms of the upper extremities for at least 12 months, with a predominance of low-intensity in the shoulder (55.5%). In addition, 88.8% reported pain in other segments. Muscle strength of abduction (rho = 0.49), adduction (rho = 0.40), internal rotation (rho = 0.44) and hand grip (rho = 0.68) presented moderate correlation with FIT-HaNSA. Hand grip (rho = − 0.52) showed moderate correlation with upper extremity dysfunction.

**Conclusions:**

The results of this preliminary study suggested the association of shoulder strength with fatigue resistance. Also, hand grip strength was associated with upper extremity dysfunction and fatigue resistance. No association was found with the Work Ability Index in this sample. So, it is suggested that hand grip and shoulder strength could be outcome measurements used for future interventions focused on upper extremity preventive exercises to improve strength and fatigue resistance of workers at risk for the development of musculoskeletal disorders. Other individual, psychosocial and organizational risk factors must also be considered as influences on upper extremity function.

## Highlights

- Shoulder abduction, adduction and internal rotation strength were associated with the upper extremity fatigue resistance of workers
- Hand grip strength was inversely correlated to upper extremity dysfunction in workers
- Upper extremity muscle strength was not associated with the ability to work
- Hand grip and shoulder strength could be outcome measurements for future interventions focused on preventive exercises to improve the upper extremity strength and fatigue resistance of workers

## Background

Upper extremity musculoskeletal disorders affect productivity, resulting in disability and absenteeism. They can be multifactorial and related to occupational and non-occupational risk factors [6, 16]. Physical demands involving repetitive work, lack of muscle recovery, precision of movements and static postures, as well as psychosocial factors, are important risk factors for the development of upper extremity disorders among females and males [30, 39, 46, 52, 56].

Overall, there is a substantial prevalence (36.8%) of work-related upper extremity complaints [7, 31]. These complaints show a high prevalence in different populations of hospital workers, reaching 62.1% for shoulders and 51.7% for wrists and hands in nurses in an orthopaedic sector [14]. For workers in a hospital nutrition service, a prevalence of 29% was found for complaints in the wrist and hand, 37% for the neck region and 10% for the elbow joint [24], and 70.1% of hospital cleaning service workers had musculoskeletal complaints [8]. Since musculoskeletal pain in the upper extremity represents a relevant prevalence, it becomes useful to obtain more accurate information about the work demands related to this segment [25]. Thus, rehabilitation or prevention programmes, ergonomic and musculoskeletal assessments and education strategies must be considered [56].

Muscle strength is the greatest predictor of function, mobility and independence [48]. An instrument used for this measurement is the dynamometer, which can be either isometric or isokinetic. Isokinetic evaluation analyses strength, power, work, fatigue percentage and muscle performance and classifies these data as normal or altered. Thus, it allows for the evaluation of the effectiveness of a treatment, dictates the rehabilitation objectives and establishes normative values of strength [15, 18]. In addition to strength, fatigue resistance is another indicator of functionality and physical capacity. Work demand is an important ability to be analysed, considering that muscle fatigue is a complaint frequently reported among workers [52]. In this context, muscle fatigue can be defined as an imbalance between work demands and the worker’s ability to perform a task, and it is associated with a decrease in motor performance, speed and range of motion and may be caused by repetition of a single activity for prolonged periods [54]. Long-term fatigue increases the risk of incapacity for work and impairs the ability to maintain a satisfactory level of production [52, 59].

Recent studies with the objective of evaluating the effectiveness of a multimodal intervention programme through specific resistance exercises, muscle stretching, massage, education and ergonomic guidelines carried out in the workplace for reducing musculoskeletal pain symptoms in the upper extremity and neck have shown promising results [1, 33, 42, 45, 47, 49, 56, 60].

Considering that work-related upper extremity dysfunction is a growing issue, showing high prevalence, high costs, long-term absenteeism and decreased productivity in different economic sectors [31, 56], it is necessary to develop studies that analyse relevant factors for future preventive or therapeutic approaches directed to specific population needs. Thus, to gain a better understanding and define the best primary outcome variables involved in workers’ musculoskeletal complaints for future interventions, the aim of this study was to analyse the association of the muscle strength of the shoulder, elbow and hand with fatigue resistance, work ability and upper extremity dysfunction in a sample of workers at a university hospital. Thus, with this preliminary study, we propose an evaluation protocol for a randomized clinical trial to be implemented in a tertiary hospital, which aims to compare the effect of ergonomic guidelines associated or not with upper extremity strengthening workplace exercises.

## Methods

This cross-sectional observational study was approved by the local Research Ethics Committee (No. 2,724,782, CAAE 89138818.1.0000.5440) in accordance with the 1964 Declaration of Helsinki and its subsequent amendments. This study was part of a randomized controlled trial (www.ClinicalTrials.gov NCT04047056). All participants signed an informed consent form before participating.

### Sample

The sample size calculation was performed by a priori analysis with G-Power 3.1 (Axel Buchner, Germany) [17], based on the rationale of difference of shoulder abduction strength between symptomatic x asymptomatic upper extremity (alpha 0.05, power 0.8 and effect size 0.5) and identified 27 participants. The sample consisted of workers from a tertiary university hospital invited to participate in this cross-sectional study. The participants were people of both sexes, aged between 25 and 60 years, who could have had pain or discomfort in the upper extremity over the previous 12 months, which may have been unilateral or bilateral, classified with a predominance of static or dynamic muscle contraction work of the upper extremity during labour activities. Dynamic work was characterized as alternating postures with consequent alternation of contraction and relaxation of the muscles of the upper extremities during a work cycle. Static work was characterized as a predominance of isometric muscle contraction with static or sustained postures and use of prehension for prolonged periods of time [16, 28]. We excluded people who had recent surgery or trauma and were unable to perform the proposed tests for physical or cognitive reasons.

### Outcomes

#### Numerical pain rating scale (NPRS)

Using an 11-point scale ranging from 0 (no pain) to 10 (worst pain ever), participants were instructed to choose the numerical value that best represented their pain intensity at that moment. A rating was made as follows: 0, no pain; 1–3, mild pain; 4–6, moderate pain; and 7–10, severe pain [20].

#### Lateral preference inventory

To assess laterality, the Lateral Preference Inventory was applied to analyse manual preference in tasks representative of the ADLs. It is divided into eight items and the participants fill in the activities according to their preferences in the options: always left, left majority, indifferent right majority, always right and I don’t know. At the end, the participants are classified as right-handed, left-handed or ambidextrous [34].

#### Nordic musculoskeletal questionnaire

This questionnaire was used to define the location and chronicity of symptoms. It consisted of a body map divided into nine segments: neck, shoulders, upper back, elbows, wrists/hands, lower back, hips/thighs, knees and ankles/feet and presented four questions with binary responses (“yes/no”) for each segment [29].

#### International physical activity questionnaire (IPAQ)-short version

This instrument was applied in order to estimate the weekly time spent in physical activities, taking into account walking activities and activities with moderate and vigorous intensity that had a minimum duration of 10 continuous minutes, in addition to the time spent in the sitting position. The participants were characterised as very active, active, irregularly active or sedentary [37].

### Quick-disabilities of the arm, shoulder and hand (QuickDASH-Br)

This questionnaire was used to define the level of upper extremity dysfunction. It is composed of 11 items, taken from the DASH questionnaire of 30 items, designed to assess the symptoms and physical and social functions related to complaints in the upper extremity. Each item was scored on a scale from 1 to 5 points, where 1 indicated “no difficulty” and 5 indicated “extreme difficulty”, with a final score ranging from 0 to 100 and the maximum score indicating greater dysfunction in the upper extremity [5, 10].

#### Work ability index

This instrument was used to assess, through the worker’s self-report, the participants’ perceptions of working conditions and physical, mental and social capacities that may be related to their complaints. The index contains 10 questions divided into seven domains and the results provide a measure of work ability, which range from 7 to 49 points, and are classified by ability as low (7 to 27), moderate (28 to 36), good (37 to 43) or excellent (44 to 49) [35].

#### Isokinetic and isometric dynamometer

Muscle strength was assessed using the Biodex System 4 Pro™ (Biodex Medical Systems, Inc., Shirley, NY, USA) isokinetic dynamometer following all the calibration and use recommendations proposed by the manufacturer in the manual. The force variable used was the mean torque peak (concentric mode), at a speed of 60°/sec as recommended for the evaluation of orthopaedic complaints [44].

**Fig. 1:**
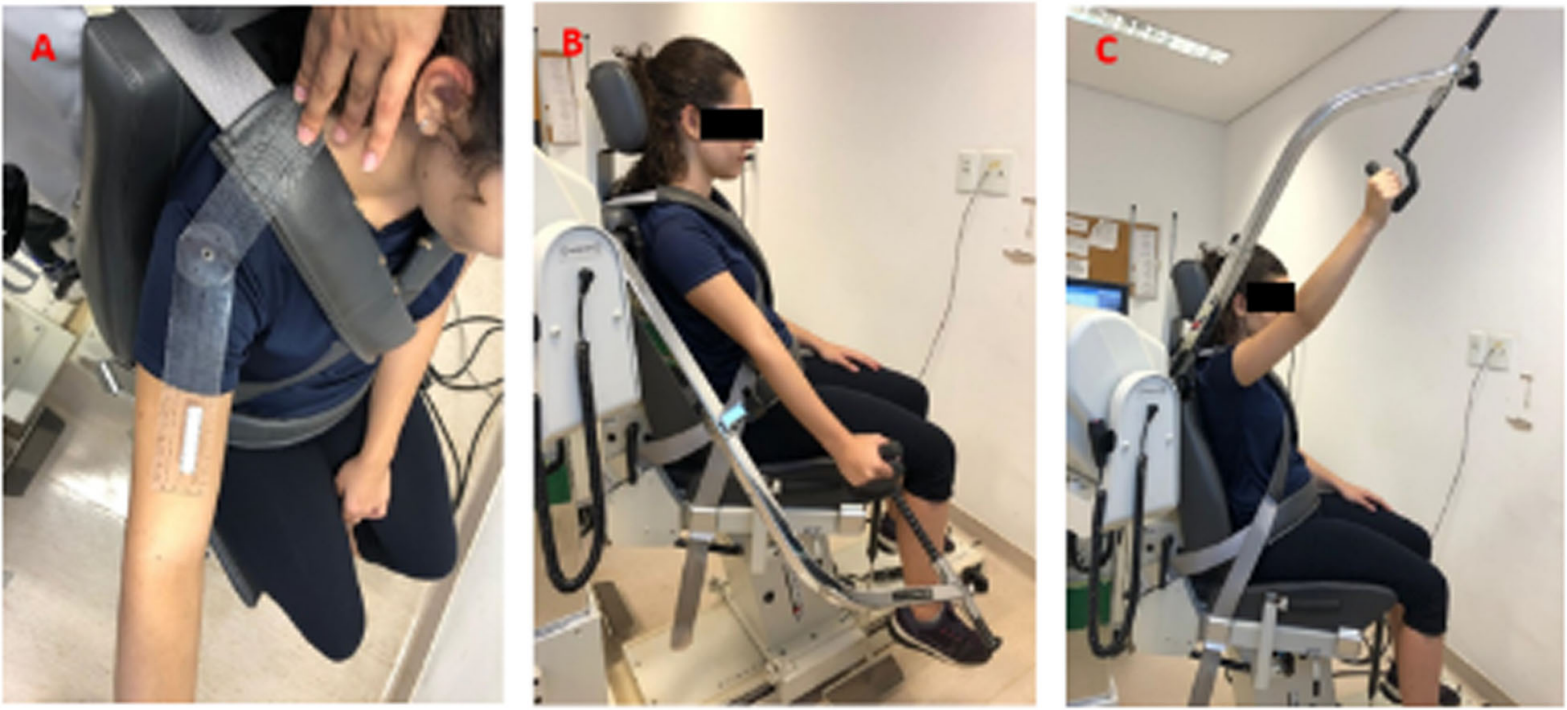
**a**, **b** and **c** Shoulder in the scapular plane to assess abduction and adduction

The participant’s position was based on the guidance material provided by the Biodex™ system. The movements evaluated were abduction/adduction of the shoulder in the scapular plane [27, 38], positioning the shoulder at 30° anterior to the frontal plane (Fig. 1a), in a range of motion from 30° (Fig. 1b) to 120° (Fig. 1c).

**Fig. 2:**
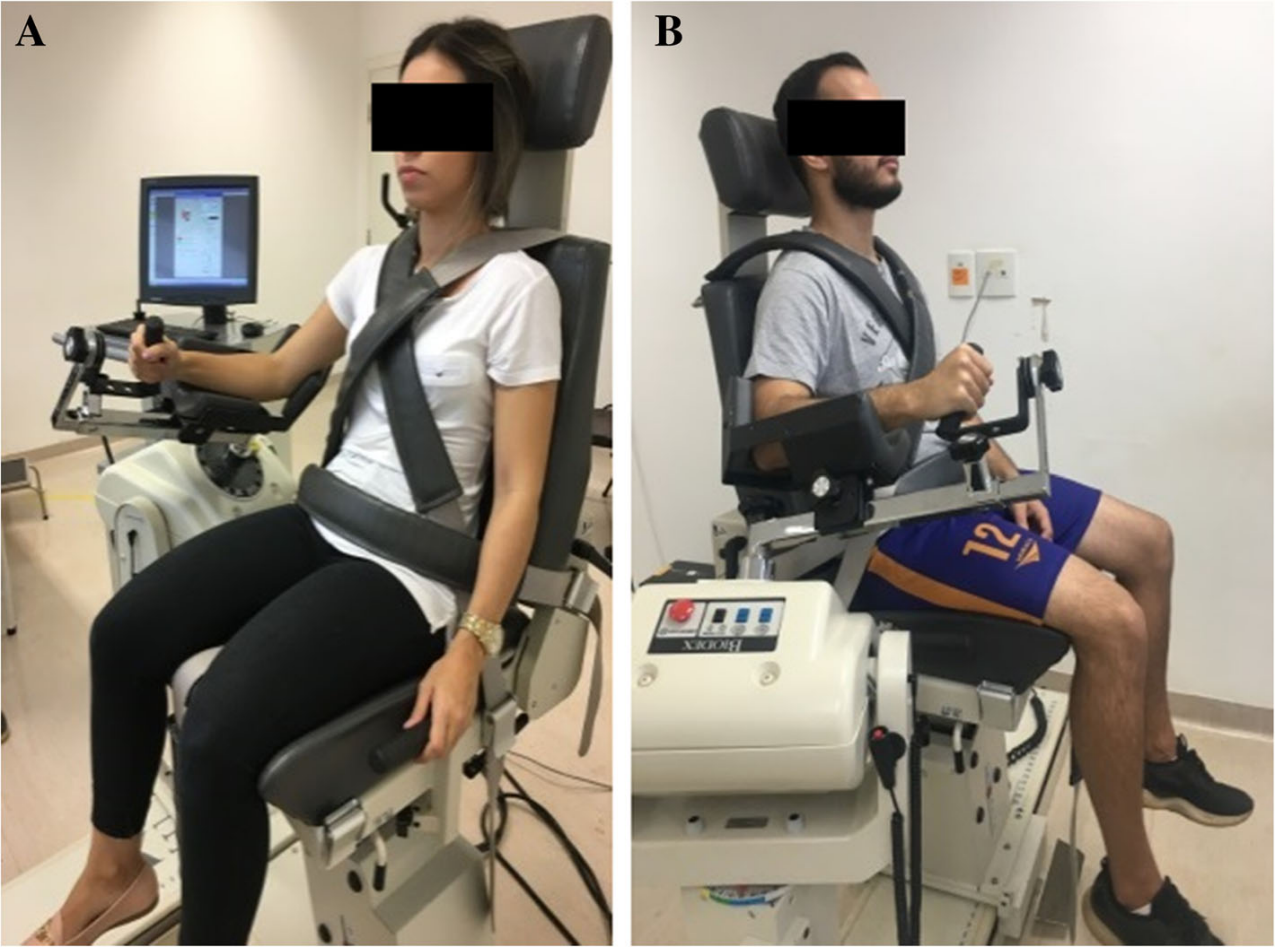
**a** and **b** Shoulder position to assess internal and external rotations

**Fig. 3:**
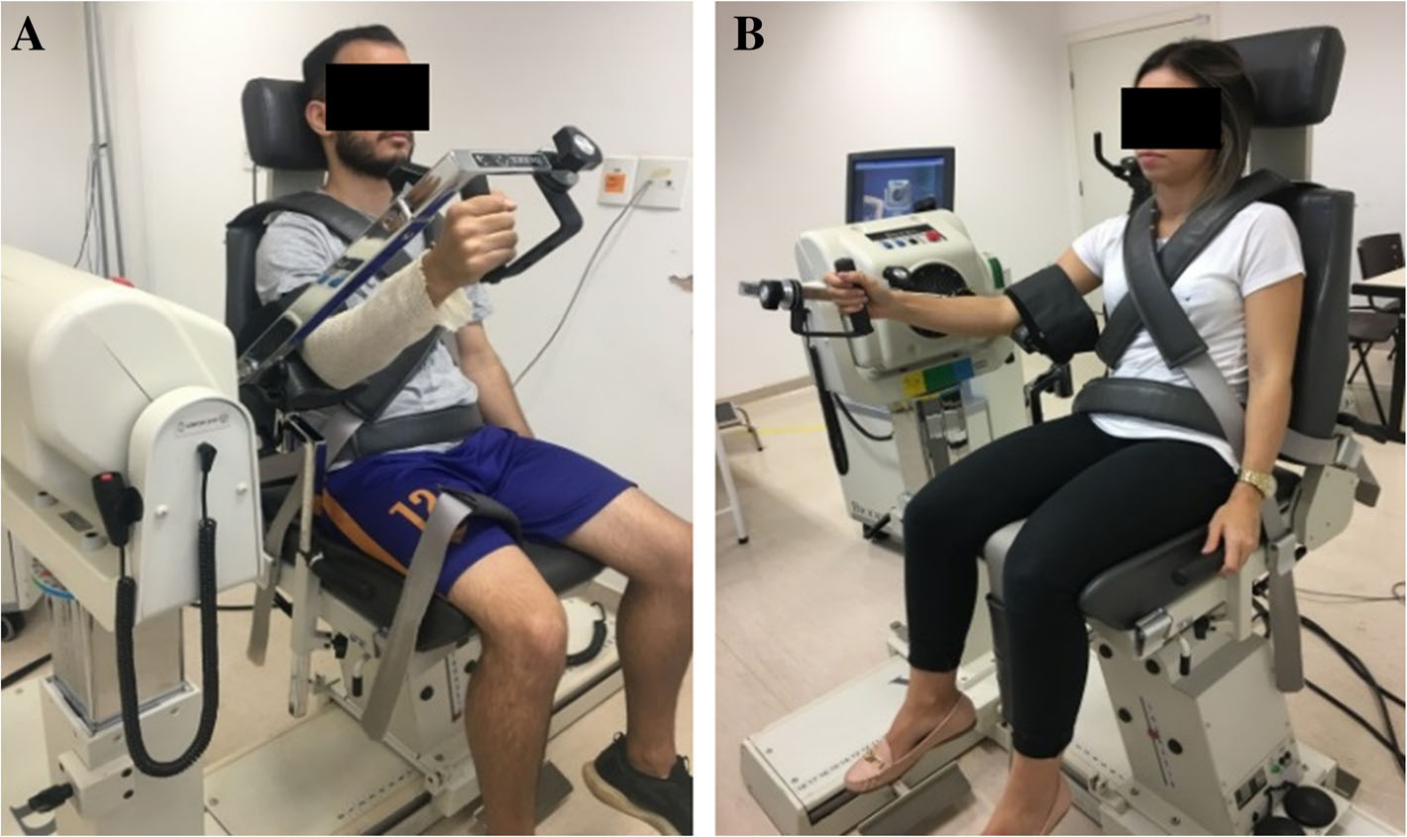
**a** and **b** Elbow position to assess flexion and extension

The internal and external rotations of the shoulder were also assessed in a range of motion from 30° (Fig. 2a) to 30° (Fig. 2b). Isokinetic elbow flexion and extension were assessed in a range of motion from 0° (Fig. 3b) to 130° (Fig. 3a). Five maximal repetitions were performed for each movement, and the asymptomatic extremity was tested first and then the symptomatic extremity. The evaluator stimulated the production and maintenance of strength through the verbal command “strength, strength, strength” during the effort.

**Fig. 4:**
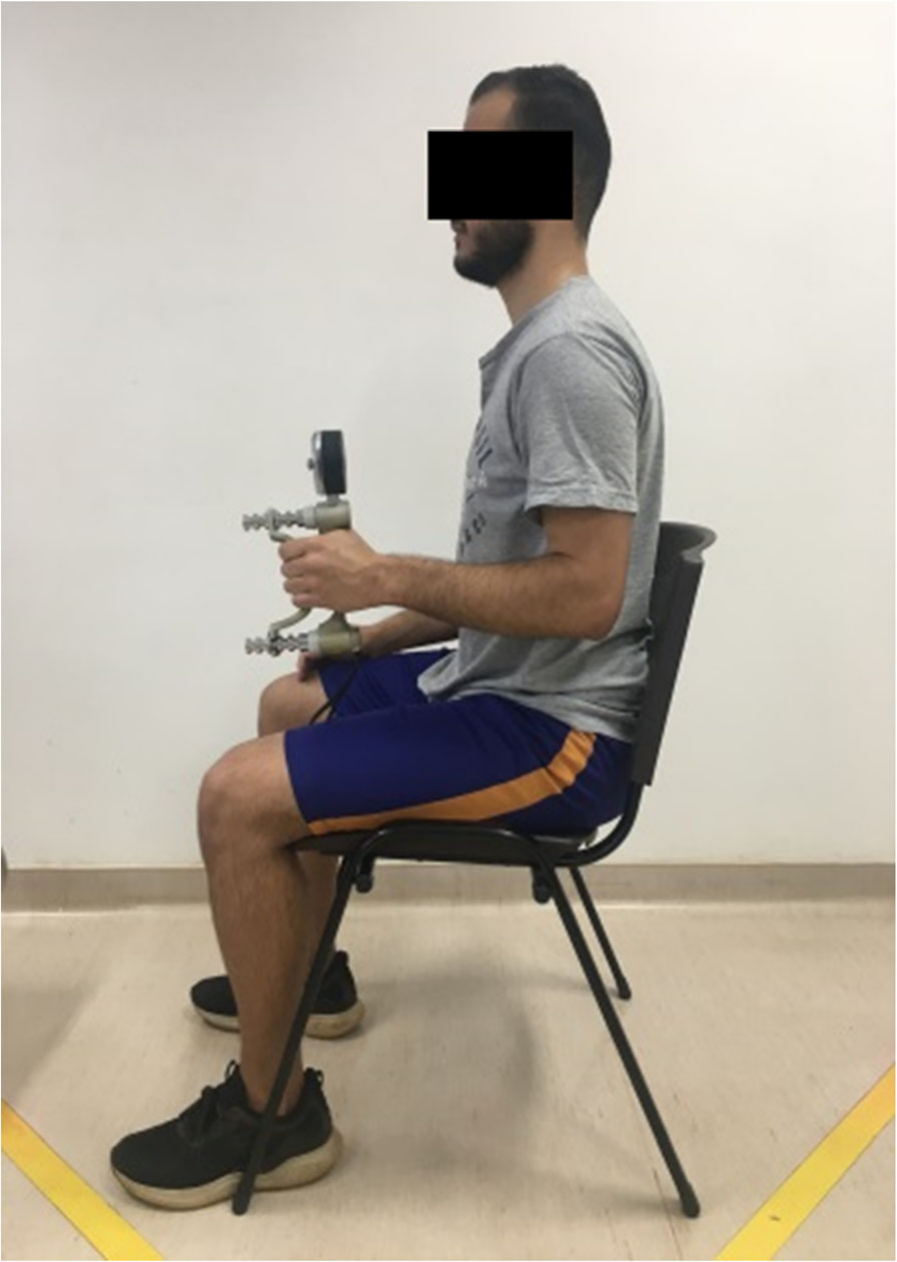
Position to assess handgrip

Hand grip strength was assessed by a JAMAR™ isometric dynamometer (National Institute for Health Research, Southampton), which is an instrument recommended by the American Society of Therapists of Hand (ASTH). The participant was placed seated in a chair without an armrest, their feet flat on the floor, shoulder adducted with neutral rotation, elbow flexed at 90° and the forearm in a neutral position (Fig. 4). The JAMAR™ handle was attached in the second position, which allows a balanced activation of the intrinsic and extrinsic flexors of the fingers [43]. Three maximal repetitions were performed on each side starting from the asymptomatic upper extremity. The time for the execution of each attempt was on average 5 s, and the alternation between one member and another was the time established for rest. The evaluator stimulated the production and maintenance of strength through the verbal command “strength, strength, strength” during the effort. At the end, the average of the three measurements was calculated.

### Fatigue resistance

**Fig. 5:**
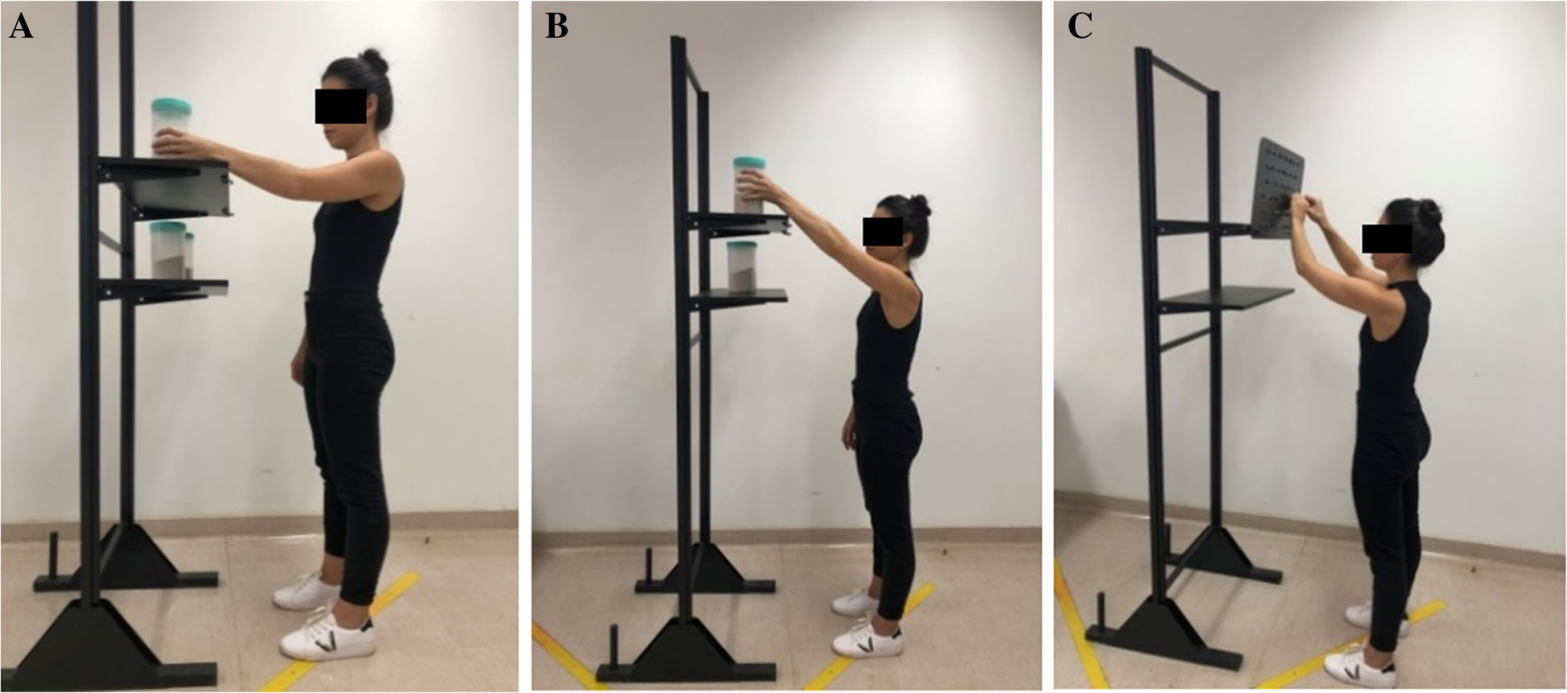
**a**, **b** and **c** FIT-HaNSA - Task 1, Task 2 and Task 3

A JobSim™ System (JTech medical industries, Inc.,USA) prototype was developed and used to perform the upper extremity fatigue resistance test, or Functional Impairment Test-Hand, and Neck/Shoulder/Arm (FIT-HaNSA). It is a three-task test that represents upper extremity gross motor functions, such as reaching and holding objects at different heights and work sustained above the head [32] (Fig. 5). The test consists of performing two unimanual tasks, performed by both sides, starting with asymptomatic side and one bimanual task developed in the following steps:

- *Task 1) Waist-Up:* One shelf was placed at waist level and the other 25 cm above it. Three 1 kg containers were placed on the lowest shelf. The participant was instructed to move the three containers from one shelf to another, at a speed of 60 beats/minute, controlled by a metronome.
- *Task 2) Eye-Down:* One shelf was placed at eye level and the other 25 cm below it. The participant was again instructed to move the three containers from one shelf to another at the same speed (60 beats/minute).
- *Task 3) Overhead-Work:* A board containing screws was attached perpendicularly to the shelf at eye level. The participants were instructed to keep both arms raised and use them to screw and unscrew the screws in a predetermined sequence: the screw that was on level 1 (top) must be moved to level 2 (middle), the screw from level 3 (lower) to level 1 and the screw from level 2 to level 3.

Each task was performed only once for a maximum of 300 s or when the participant interrupted the test due to pain, upper extremity fatigue or disability. The rest between one task and another was established by the time taken to adjust the shelf for the subsequent task [32]. To analyse the correlations, the average time of the three tasks was calculated.

### Procedure

The evaluation was carried out in a single day and applied by the same physiotherapist. If the participant had bilateral complaints or pain in more than one segment of the upper extremity, the site and member of highest pain intensity was defined as the symptomatic member/site measured by the NPRS.

Before starting the tests, instrument familiarisation and randomisation of upper extremity muscle strength and fatigue resistance tests were performed. The tests were preceded by a 5 min warm up on the cycle ergometer and as a rest criterion, a sociodemographic form and the questionnaires were applied.

### Statistical analysis

The associations between the mean values of strength, fatigue resistance, work ability and dysfunction obtained from the symptomatic upper extremity were analysed by Spearman’s correlation coefficient (*rho*). The correlation was classified as high (*r* ≥ 0.70), moderate (*r* ≥ 0.40 or *r* < 0.70) or low (*r* < 0.40) [12], adopting a 95% confidence interval. Besides that, the Wilcoxon test was applied to analyse the difference in strength between the symptomatic and asymptomatic upper extremity. The software used for statistical analysis was SPSS version 20.0™.

## Results

In all, 26 out of 27 participants were right-handed; 59.2% of participants reported pain/discomfort in the dominant upper extremity, and 40.7% of the participants reported it in the non-dominant upper extremity. A total of 37.0% of the participants reported bilateral pain, but we considered the side with the most relevant complaint for the correlation analysis. All of the participants predominantly used the upper extremities in their main functions but with different levels of effort; 62.9% of the participants performed work functions with dynamic muscle contractions characterized as “dynamic work” (cooking, cleaning, nursing and technical repair sectors). Most performed a high level of physical effort, including lifting/shifting loads with the upper extremities. The remaining 37.0% had static work characteristics with a low level of physical effort but with isometric muscle contraction maintaining the same position for prolonged periods (administrative sector and laboratory technicians).

There was a similar proportion of men and women aged between 26 and 59 years. By the IPAQ, 18.5% of the participants were classified as very active, 37.0% as active, 37.0% as irregularly active and 7.4% as sedentary. Data that characterise the sample are summarised in Table 1. Only 37% of the participants had comorbidities, and none of them were smokers.

**Table 1:** Sample characterisation

Sex - Men/Women	*n* = 11 (40.7%) / *n* = 16 (59.2%)
Mean Age (years)	46.2 SD- 8.9
BMI Classification (kg/m^2^)	Normal 18.5–24.9^a^ *n* = 8 (33.3%)
	Overweight 24.9–30^a^ *n* = 7 (25.9%)
	Obese (> 30)^a^ *n* = 12 (44.4%)
Comorbidities	Arterial hypertension *n* = 4 (14.8%)
	Diabetes *n* = 2 (7.4%)
	Vascular disease *n* = 2 (7.4%)
	Rheumatoid arthritis *n* = 1 (3.7%)
	Hypothyroidism *n* = 1 (3.7%)
	Depression *n* = 1 (3.7%)

*BMI* Body mass index

*SD* Standard Deviation

^a^Thresholds values for BMI

By Nordic questionnaire, it was found that all participants had symptoms of the upper extremities for at least 12 months, with a predominance of symptoms in the shoulder (55.5%). In addition, 88.8% of the participants reported pain in other segments during the same period; 70.3% of the participants still complained of the same symptoms in the upper extremities in the last 7 days.

Pain intensity measured by the NPRS was classified as mild, and the QuickDASH-Br showed that participants had a low level of upper extremity dysfunction. In terms of FIT-HaNSA scores, a deficit of 50% in fatigue resistance was found in the symptomatic upper extremity when compared to the expected total time spent to perform the three tasks. These values are presented in Table 2.

**Table 2:** Mean values of pain intensity (NPRS), upper extremity dysfunction questionnaire (QuickDASH-Br), Work Ability Index (WAI) and upper extremity fatigue resistance Functional Impairment Test-Hand, and Neck/Shoulder/Arm (FIT-HaNSA)

	Mean	SD	(Min-Max)
NPRS	3.0	2.2	(0–7)
QuickDASH-Br	30.3	21.3	(0–61.4)
FIT-HaNSA	149.4	62.8	(43.75–266.6)
WAI	36.3	5.5	(23–46)

*SD* Standard Deviation

*FIT-HaNSA* Average time in seconds for the three tasks

*NPRS* Average pain intensity classified as mild

*WAI* Overall average work capacity classified as moderate

In relation to the Work Ability Index, 7.4% of the participants were classified as low ability, 44.4% as moderate ability, 37.0% as good ability and 11.1% as excellent ability. Still on work issues, 55.5% of the participants reported long-term absenteeism due to musculoskeletal problems.

**Fig. 6:**
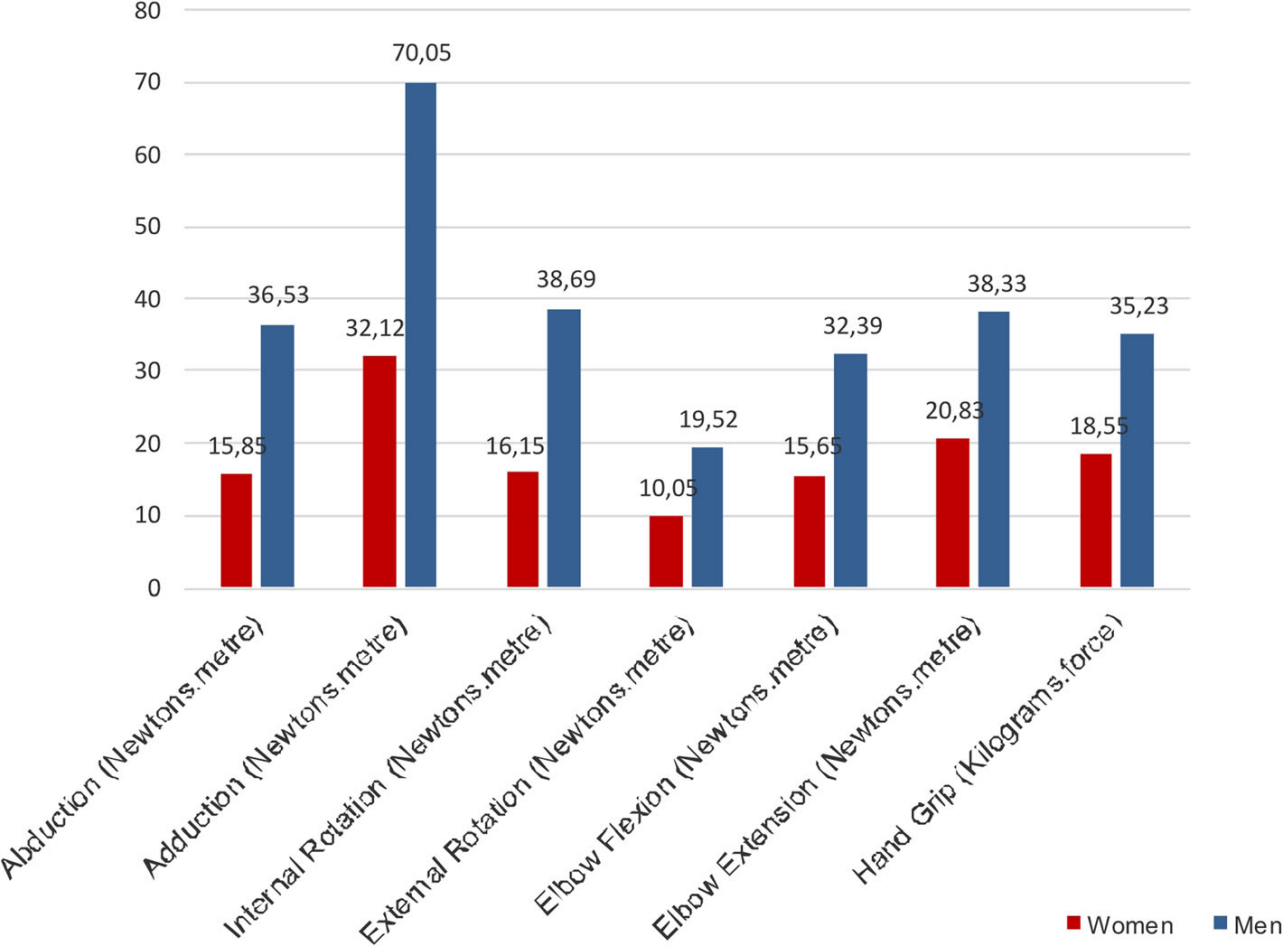
Values of strength for women and men. * Values in Newton metre (Nm) - Mean of the peak torque mean. ** Values in Kilogram.force (Kgf) - Mean of three measurements

No significant difference between the symptomatic and asymptomatic side was found for isokinetic and isometric strength muscle testing (Table 3). Mean isokinetic strength values ​​obtained for women ranged from 41.7 to 54.3% lesser than that for men. For women, the difference in hand grip strength was also 52.6% lesser than that for men (Fig. 6).

**Table 3:** Muscle strength values comparation between symptomatic X asymptomatic upper extremity with Biodex System 4 Pro™

Segment	Movement	Symptomatic/ Asymptomatic		***P*** value
Shoulder^a^	Abduction	27.19	27.57	0.30
	Adduction	46.96	53.50	0.87
	Internal rotation	28.40	24.65	0.55
	External rotation	15.68	14.75	0.59
Elbow^a^	Flexion	25.10	21.83	0.20
	Extension	32.67	36.12	0.12
Hand^b^	Hand grip	25.34	25.87	0.87

^a^ Values in Newton metre (Nm) - Mean of the peak torque mean

^b^ Values in Kilogram.force (kgf) - Mean of three measurements

Muscle strength showed a significant correlation with FIT-HaNSA, except regarding to shoulder external rotation and elbow extension, with positive and moderate correlations for abduction, adduction, internal rotation and hand grip. Hand grip strength also showed a moderate negative correlation with upper extremity dysfunction. Table 4 shows the correlations obtained and the respective statistical significance level considering a 95% confidence interval (*p* < 0.05).

**Table 4:** Values of Spearman correlation coefficient (rho) of upper extremity muscle strength with fatigue resistance test and self-report questionnaires

Mean Torque Peak	QuickDASH***-***Br	Work Ability Index	FIT-HaNSA
**Shoulder**			
Abduction	−0.34	0.19	0.49
	0.08	0.34	0.01*
Adduction	−0.18	−0.01	0.40
	0.36	0.92	0.03*
Internal Rotation	−0.32	0.10	0.44
	0.09	0.58	0.02*
External Rotation	−0.36	0.14	0.33
	0.06	0.48	0.08
**Elbow**			
Flexion	−0.35	0.20	0.38
	0.07	0.31	0.05*
Extension	−0.05	−0.18	0.24
	0.80	0.3	0.22
**Hand grip**	−0.52	0.31	0.68
	0.00	0.10	0.00*

*FIT-HaNSA* Functional Impairment Test-Hand, and Neck/Shoulder/Arm

* Significant associations (*p* < 0.05)

## Discussion

Our data showed a significant and moderate association between upper extremity fatigue resistance for all shoulder muscle strength movements, except for external rotation. We also observed a significant and moderate correlation between hand grip strength and upper extremity dysfunction. It is known that hand grip strength is correlated with different functional disorders of the upper extremity, such as rheumatoid arthritis, carpal tunnel syndrome, lateral epicondylalgia, stroke, traumatic injuries and neuromuscular diseases, and it is also strongly related to general health status [22, 40]. The sample of our study presented only one worker with upper extremity dysfunction related to rheumatoid arthritis, which was asymptomatic. To be included in the sample, they could have had pain or discomfort in the upper extremity over the previous 12 months, and for appraisal, the most affected side was considered. It is known that musculoskeletal pain can aggravate the development of muscle fatigue. A study observed that resistance training significantly improved muscle strength and reduced pain and disability, suggesting an association between fatigue resistance and strength in a sample of workers complaining of chronic pain in the upper extremities [53].

Even with a low level of upper extremity dysfunction and a satisfactory level of physical activity of this sample, these characteristics might not be enough for most participants to perceive that they had excellent ability to work. There was no correlation between upper extremity muscle strength and ability to work. A poor association between physical performance and work ability has also been observed by other authors [51]. This can be explained by the fact that the ability to work is not only influenced by physical function but also by psychological, cognitive and social functioning. These risk factors are, in turn, affected by pain, and the association of these variables negatively interferes with the ability to work [23, 51].

Although mostly active, 70.3% of the sample was overweight or obese according to their BMI. Some studies suggest that pain and obesity are correlated, and this relationship can be measured by factors such as age, structural changes, inflammatory chemical mediators and mood and sleep disorders, all of which are potential markers of functional and psychological complications in chronic pain [2, 21, 41]. There seems to be a consensus in the literature that, when maximum muscle strength is normalised to body mass, obese individuals are weaker compared to non-obese individuals, especially in relation to the anti-gravitational muscles [55]. This condition of decreased muscle strength can increase the risk of developing osteoarthritis and lead to functional limitations, especially in the elderly. Evidence also suggests that high levels of adiposity may impair agonist muscle activation in the young, adding to or leading to the functional limitation [55]. A longitudinal study that followed kitchen workers showed that obesity was a risk factor for generalised pain [21]. In addition, self-reported pain at different body segments may increase the risk of absenteeism and disability [21, 36]. These studies agree with our data sample, of which 55.5% of the participants reported long-term absenteeism due to musculoskeletal complaints. Musculoskeletal disorders in healthcare professionals and other workers are highly prevalent and associated with workload and ergonomics factors [13, 50].

The use of self-report questionnaires allied to performance tests can be useful as outcome measurements, which could form the baseline for clinical trials to address musculoskeletal conditions [3, 11]. Shoulder strength and hand grip strength were associated with the resistance to the upper extremity fatigue test (FIT-HaNSA) and can be applied in workers as dynamic and static muscle tasks [4]. Nevertheless, many different factors such as glenohumeral elevation angles, exertion velocity [38] and body position [19] could interfere with results.

Our sample had a balance of men and women, and the results showed that women had almost half the strength obtained by men. These data corroborate with previous studies that observed that most of the muscle groups evaluated in men were 1.5–2 times stronger than those in women [9]. A study that assessed normative clinical data for upper extremity strength of men and women, found significantly higher strength values in men for all muscle groups evaluated in the upper extremities, and also observed that the dominant side was stronger than the non-dominant side in both men and women [57]. In our study, there was no significant difference in strength between the upper extremities. This may have occurred because the majority of the complaints of the study participants were in the dominant extremity.

### Study limitations

Many issues may limit the ability to generalise these findings. The methodology used does not allow us to establish a predictive factor for upper extremity musculoskeletal complaints and muscle strength changes. In addition, the sample size did not allow the analysis of subgroups that would consider the individuality of each worker profile in terms of magnitude and characteristics of effort, besides from being a factor that could overestimate the findings of the study. There were also no detailed analyses of the association of self-reported pain and ergonomic conditions of the work environment or on other risk factors, which would be relevant, considering that the ability to work could be influenced by these aspects [7, 58].

Our findings suggested that hand grip and shoulder abduction and adduction isokinetic strength could be outcome measurements of particular relevance for the development of assessment protocols for pre-interventions focused on prevention of the development of musculoskeletal complaints in the upper extremity related to work, through muscle conditioning exercises to improve upper extremity strength and fatigue resistance.

Individual, psychosocial and organizational risk factors must also be considered to influence upper extremity physical dysfunction and ability to work. A multifaceted approach focused on improving physical conditions, as well as psychosocial/organizational, ergonomics and environmental aspects of work must be addressed [26, 58].

## Conclusion

Shoulder abduction, adduction and internal rotation strength were associated with upper extremity fatigue resistance. Also, hand grip strength was associated with upper extremity dysfunction and fatigue resistance. No association was found with the to Work Ability Index in this sample. In this way, it is suggested that hand grip and shoulder strength could be outcome measurements to be used for future interventions focused on preventive exercises to improve strength and fatigue resistance of the upper extremities of workers at risk for the development of musculoskeletal disorders. Other individual, psychosocial and organizational risk factors must also be considered as influences on upper extremity physical function.

## Data Availability

The datasets used and/or analysed during the current study are available from the corresponding author as additional files.

## Abbreviations

ADLs: Activities of daily living
ASTH: American Society of Therapists of Hand
BMI: Body Mass Index
DASH: Disabilities of the Arm Sholder and Hand
FIT-HaNSA: Functional Impairment test Hand/Neck/Shoulder and Arm
IPAQ: International Physical Activity Questionnaire
Kgf: Kilogram Force
Nm: Newton Metre
NPRS: Numeric Pain Rating Scale
SD: Standard Deviation
WAI: Work Ability Index
